# LARS‐augmented hamstring ACL reconstruction shows better early but similar long‐term outcomes compared with hamstring autograft alone: A systematic review and meta‐analysis

**DOI:** 10.1002/jeo2.70654

**Published:** 2026-02-24

**Authors:** Panagiotis Antzoulas, Vasileios Giannatos, Andreas Panagopoulos, John Lakoumentas, Vasileios Athanasiou, Antonios Kouzelis, Zinon Kokkalis, Irini Tatani, Spyridon Plesas, John Gliatis

**Affiliations:** ^1^ Department of Orthopaedics University Hospital of Patras Patras Greece; ^2^ Department of Medical Physics, School of Health Sciences University of Patras Patras Greece; ^3^ Department of Orthopedics General Hospital of Nikaia Athens Greece

**Keywords:** ACL reconstruction, hamstring autograft, LARS, ligament augmentation, return to sport

## Abstract

**Purpose:**

This systematic review aimed to evaluate whether augmenting hamstring autografts with the Ligament Augmentation and Reconstruction System (LARS) during anterior cruciate ligament reconstruction (ACLR) enhances early recovery, improves functional outcomes, and accelerates return to sport (RTS), compared to standard hamstring autografts alone. With increasing emphasis on accelerated rehabilitation, the study seeks to clarify the clinical role of synthetic graft augmentation.

**Methods:**

Following the PRISMA (Preferred Reporting Items for Systematic reviews and Meta‐Analyses) guidelines, a comprehensive systematic review was conducted focusing on ACLR using hamstring autografts augmented with LARS. The protocol was registered on PROSPERO (CRD42024536835). A literature search was performed using PubMed and Scopus databases. Primary outcome was anterior laxity based on KT‐1000, while secondary outcomes included the Lysholm score, Tegner activity scale, Knee injury and Osteoarthritis Outcome Score (KOOS), International Knee Documentation Committee (IKDC), anterior cruciate ligament‐return to sport after injury (ACL‐RSI), complication rates, graft failure and RTS. Methodological quality was assessed using the Newcastle–Ottawa Scale. Six studies involving a total of 764 patients met the inclusion criteria.

**Results:**

LARS‐augmented ACLR was associated with significantly better early functional outcomes, including higher Lysholm, IKDC and Tegner scores (standardized mean difference [SMD] = 0.83, 0.5 and 0.72, respectively), higher psychological readiness (ACL‐RSI) and earlier RTS. KT‐1000 measurements showed no statistically significant difference between the hybrid and autograft groups. However, long‐term outcome differences between augmented and non‐augmented groups were minimal.

**Conclusion:**

LARS augmentation in ACLR is a promising technique, supporting early rehabilitation, secure graft stability and timely return to activity, potentially benefiting older individuals or those with higher body mass index (BMI). Selective use of LARS may be advantageous in high‐demand patients or early RTS protocols, while future implications could include augmentation in case of suboptimal graft quality.

**Level of Evidence:**

Level III.

**Abbreviations**
ACL: anterior cruciate ligament
ACLR: anterior cruciate ligament reconstruction
ADL: activities of daily living
HT: hamstring tendon
IKDC: International Knee Documentation Committee
KOOS: Knee injury and Osteoarthritis Outcome Score
LARS: Ligament Augmentation and Reconstruction System
PRISMA: Preferred Reporting Items for Systematic reviews and Meta‐Analyses
PROSPERO: International Prospective Register of Systematic Reviews
QOL: quality of life
RCT: randomized controlled trial
RoB: risk‐of‐bias
RSI: return to sport after injury
RTS: return to sport
SMD: standardized mean difference

## INTRODUCTION

Anterior cruciate ligament (ACL) rupture is the most common ligamentous injury of the knee in athletes and active individuals [20]. A major motivation for patients undergoing ACL reconstruction (ACLR) is the desire to return to sport (RTS) as early as possible [4]. However, despite evolving rehabilitation protocols targeting accelerated RTS, substantial deficits in strength, neuromuscular control, and graft maturation often persist for up to 12 months postoperatively, contributing to increased failure rates when RTS occurs prematurely [5, 25].

Hamstring autografts remain one of the most widely used options for ACLR due to their ease of harvest and favourable donor‐site morbidity profile [37]. Nonetheless, RTS outcomes remain suboptimal: fewer than two‐thirds of patients return to their preinjury level, only slightly more than half ultimately resume sports participation, while for those returning, a minimal time frame of 9–12 months has to be followed despite recent efforts with suture tape augmentation techniques [2, 8, 17, 24]. Graft failure and recurrent instability remain key concerns, influenced by graft choice, surgical technique, concomitant procedures and rehabilitation strategy [11, 17].

Early graft remodelling represents a vulnerable period [36]. During the first 5–9 weeks post‐ACLR, the graft undergoes hypocellular and hypovascular changes and remains biomechanically weak before revascularization and synovialization progress [27, 35, 36]. This biological window increases susceptibility to graft stretching and failure, supporting guidelines that restrict pivoting or high‐impact activities for at least 3–4 months following surgery [28]. Evidence on accelerated rehabilitation remains mixed: while it may facilitate earlier functional recovery, several randomized trials suggest it may compromise graft stability [3, 7, 10, 12, 26, 29].

While the four‐stranded hamstring autograft remains the workhorse of ACLR throughout the years, research into allografts revealed numerous complications, as did early research on synthetics [1, 21, 33, 37]. Synthetic materials were first introduced for ACL reconstruction in the 1970s, offering high tensile strength, no donor‐site morbidity and no risk of disease transmission [33, 37]. However, early generations were associated with high failure rates, synovitis and joint effusion, leading to widespread abandonment [18]. In recent years, synthetic augmentation—particularly the Ligament Augmentation and Reconstruction System (LARS)—has re‐emerged as a means to protect soft‐tissue grafts, reduce early laxity, and support earlier functional recovery [18]. Modern LARS constructs are designed to be biocompatible and facilitate cellular ingrowth, avoiding many complications reported with older synthetic grafts [18, 34].

A recent systematic review by Zhao et al. examined outcomes of hamstring autografts with LARS augmentation [40], but added the first study from Zaid et al [38]. However, their review omitted the newer study by Zaid et al. [37], which found no significant clinical differences beyond 18 months between hybrid and hamstring‐only grafts. Additionally, the inclusion of a study by Liu et al. comparing stand‐alone synthetic grafts to autografts may have introduced bias, as fully synthetic grafts exhibit distinct biomechanical properties—particularly increased early postoperative stiffness—that differ from hybrid constructs [22, 23].

The aim of this systematic review is to compare functional outcomes between ACL reconstructions using hamstring autografts augmented with LARS and those using hamstring autografts alone. The primary outcome is postoperative anterior knee laxity measured using the KT‐1000 arthrometer. Secondary outcomes include graft failure rates, RTS timelines, and patient‐reported outcome measures (PROMs), including International Knee Documentation Committee (IKDC), Knee injury and Osteoarthritis Outcome Score (KOOS), Lysholm, Tegner and ACL‐RSI scores.

## MATERIALS AND METHODS

### Study design and protocol registration

This study adhered to the Cochrane methodology for systematic reviews [3]. The predefined protocol was published in PROSPERO (International Prospective Register of Systematic Reviews; Registration Number CRD42024536835). The research was conducted in alignment with the PRISMA (Preferred Reporting Items for Systematic reviews and Meta‐Analyses) guidelines and the Knee Surgery Sports Traumatology Arthroscopy (KSSTA) guidelines for reporting of systematic reviews [29, 30]. Two independent researchers systematically searched international databases, including PubMed and Scopus, for relevant English‐language literature up to 4 September 2024. Differences between researchers were resolved through discussion, with consultation from a third reviewer when necessary. The detailed search strategy used is listed in Supporting Information S1: Appendix S1, with no restrictions on publication years.

### Search strategy

The search strategies combined specific keywords with the Boolean operator ‘AND’ as follows: in PubMed, ‘(((ACL) OR (ANTERIOR CRUCIATE LIGAMENT)) AND (RECONSTRUCTION)) AND (LARS)’, and in Scopus, ‘(“acl” OR “anterior cruciate ligament”) AND “reconstruction” AND “LARS”’. Full‐text reviews were conducted on articles identified as relevant or where the title and abstract alone were insufficient for an inclusion decision. Additionally, reference lists of selected studies were examined to locate any further relevant articles not captured in the initial database search.

### Eligibility criteria

The eligibility criteria included: (1) clinical studies with a prospective, retrospective, or retrospective comparative design (Levels of Evidence I, II, III or IV); (2) patients of any race or sex undergoing primary ACLR; (3) studies comparing ACL reconstruction using hamstring tendons (HTs) alone versus HTs with LARS augmentation; (4) evaluation of outcomes such as patient‐reported outcomes measurements (PROMs), knee laxity, complications, return‐to‐sport rates, and graft failure rates and (5) a minimum follow‐up period of 1 year.

Studies that did not meet the inclusion criteria were excluded. The exclusion criteria encompassed case reports, review articles, editorial letters, expert opinions and non‐comparative studies. Additionally, studies that lacked clear data relevant to our analysis, had a follow‐up period shorter than 1 year, or were based on biomechanical, cadaveric or animal models were not considered. Finally, articles not available in English were excluded also.

### Data extraction and outcomes

Data were independently extracted by two reviewers using a standardized form. Extracted data included:

- First author, publication year, country, study design
- Patient demographics and sample size
- Graft type and surgical technique
- Type of LARS augmentation
- Outcome measures and follow‐up duration

The primary outcome was anterior knee laxity (KT‐1000). Secondary outcomes included PROMs (IKDC, KOOS, Lysholm, Tegner, ACL‐RSI), complication/failure rates and RTS percentages.

### Quality assessment

To assess the methodological quality and risk of bias (RoB) of the included studies, different evaluation tools were employed based on study design. Five studies were categorized as cohort and the Newcastle–Ottawa Scale (NOS) was utilized, which evaluates studies based on selection, comparability and outcome domains [16, 31]. One study was categorized as randomized controlled trial (RCT) and the RoB2 tool was used for assessment [16, 32]. Two independent reviewers (P. A. and V. G.) performed the assessments, and any disagreements were resolved through discussion. Disputes were resolved through the consultation of a senior orthopaedic surgeon (A. P.).

### Study selection

**Figure 1:**
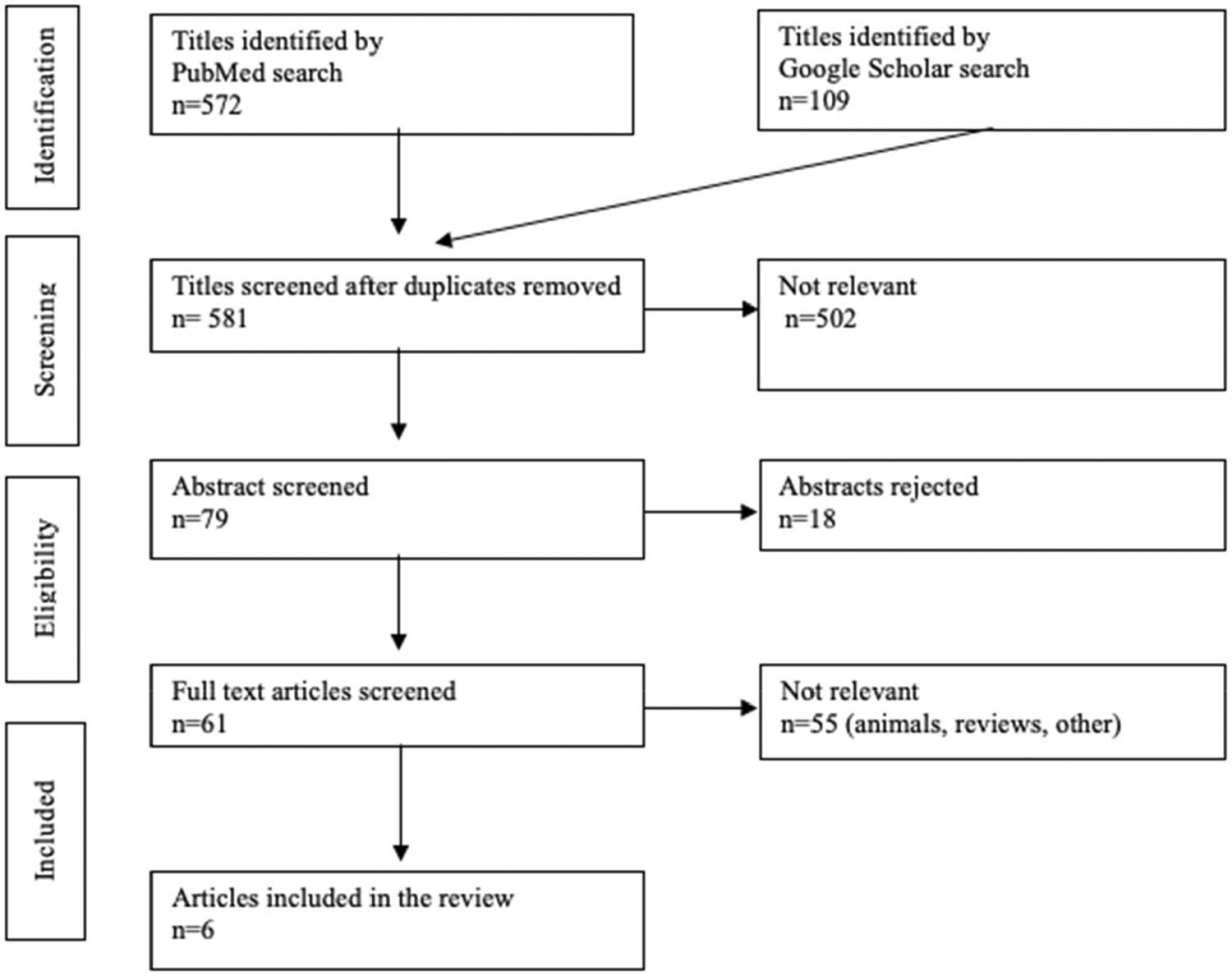
Flowchart of the article selection process. After duplicate removal, records screening and full‐text assessment, six studies were included in quantitative synthesis.

All eligible studies were written in English from 2015 to 2024 (Table 1). Six hundred and eighty‐one relevant articles were initially selected according to the search strategy. There were 581 articles left after checking for duplicates using the literature management software Mendeley. Five hundred and two articles were removed by screening the title and abstract. After reviewing the full text, eighteen articles were excluded through assessment for eligibility. Reasons for exclusion after abstract and full‐text screening included the wrong type of graft (autografts only or LARS synthetic graft instead of hybrid graft), lack of comparator group and animal studies. Eventually, six articles [4, 9, 13, 37, 38, 39] were included in qualitative and quantitative synthesis (Figure 1).

**Table 1:** Study characteristics: Detailing study type, graft choice, surgical technique and fixation methods.

Study	Study type	Patients	Group	Graft	Surgical technique	Fixation (Femur)	Fixation (Tibia)
Zaid et al. [38]	Prospective (Level II)	89/89	HT/HT + LARS	4‐SHT ST/G/4‐SHT ST/G + LARS	Single‐bundle	Endo‐Button (Smith & Nephew)	Interference screw (Smith & Nephew) and titanium staple (Citieffe)
Ebert et al. [9]	Retrospective (Level III)	69/67	HT/HT + LARS	AM: 2‐strand ST tendon; PL: 2‐strand G	Double‐bundle	Endo‐Button (Smith & Nephew)	Intra‐fix screw and Milagro advance screw (DePuy)
Zaid et al. [37]	Prospective (Level II)	47/47	HT/HT + LARS + synthetic meshwork	4‐SHT ST/G/4‐SHT ST/G + LARS + synthetic meshwork	Single‐bundle	Endo‐Button (Smith & Nephew)	Bioabsorbable interference screw (Smith & Nephew, USA) and titanium staple (Citieffe)
Aujla et al. [4]	Retrospective (Level III)	130/66	HT/HT + LARS	4‐SHT ST/G/4‐SHT ST/G + LARS	Single‐bundle	Endo‐Button (Smith & Nephew)	Intra‐fix screw (DePuy)
Zhang et al. [39]	Retrospective (Level III)	32/36	HT/LARS	2‐strand LARS‐augmented ST	Single‐bundle	Tight cord (Arthrex)	Bioabsorbable screw (Arthrex)
Hamido et al. [13]	Retrospective (Level III)	45/27	HT/HT + LARS	4‐SHT ST/G/4‐SHT ST/G + LARS	Single‐bundle	Cross‐Pin System (Mitek)	Bioabsorbable screw

Abbreviations: HT, hamstring tendon; LARS, Ligament Augmentation and Reconstruction System; SHT, strand hamstring tendon; ST/G, semitendinosus/gracilis.

### Statistical analysis

The meta‐analysis procedure was accomplished with the objective of pooling and summarizing a set of 18 outcomes/time points (such as ‘ACL‐RSI 06‐month’, etc.). For any considered outcome, the evaluated effect size was selected to be the standardized mean difference (SMD) between cases (LARS & HT) and controls (ONLY HT), for example, of the ‘ACL‐RSI’ scale. The SMDs under all circumstances are computed according to ‘Hedges’ method [14].

Per the study, one is able to compute a related SMD, its 95% confidence interval, and a *p* value of the null hypothesis that the SMD equals 0. Pooling is involved with each review separately and aims to estimate a collective SMD, its 95% confidence interval, and a pooled *p* value. For that purpose, a random effects summarizing model is applied [19]. Statistical heterogeneity is assessed via the *τ* ^2^ metric and the Sidik–Jonkman (SJ) estimator, and the *I* ^2^ metric. Also, it is evaluated via Cochrane's *Q* test, and for all of the heterogeneity metrics, a 95% confidence interval is moreover estimated. Everywhere in our study, we consider statistical significance whenever a *p* value is less than 5%. However, when summarizing study outcomes and since the number of studies is small, we also consider marginal statistical significance whenever a *p* value is less than 10% (secondary result).

As for visualizations, forest plots were used to illustrate the overall pooling of the raw proportions and the extent of heterogeneity that drove the process, while funnel plots to illustrate systematic heterogeneity and publication bias [15]. The meta‐analysis was implemented using the R statistical language (using the libraries ‘meta’, ‘metafor’ and ‘dmetar’), along with the RStudio IDE, and it was also based on the drop‐down online guidebook contained in [15]. All of the executed methods and visualizations were therefore held via verified and well‐known open‐source software tool (R‐studio).

## RESULTS

### Study characteristics

The included studies comprised one Level I prospective non‐blinded clinical trial and five Level II or III cohort studies assessing outcomes of ACL reconstruction using HT autografts with or without augmentation by LARS or synthetic meshwork (Table 1). Study designs varied, including two prospective studies [37, 38] and four retrospective cohort studies [4, 9, 13, 39]. Sample sizes ranged from 59 to 196 patients. Surgical techniques were predominantly single‐bundle reconstructions, except for Ebert's study, which utilized a double‐bundle approach [9]. Graft configurations included four‐strand semitendinosus/gracilis (ST/G) constructs, LARS‐augmented grafts, and synthetic meshwork‐enhanced grafts. Fixation methods also varied across studies, including Endo‐Button systems, interference screws, titanium staples, bioabsorbable screws and cross‐pin systems. Despite methodological differences, all studies compared HT autografts alone versus LARS‐augmented reconstructions in terms of clinical, radiographic and/or functional outcomes.

### Assessment of RoB

**Figure 2:**
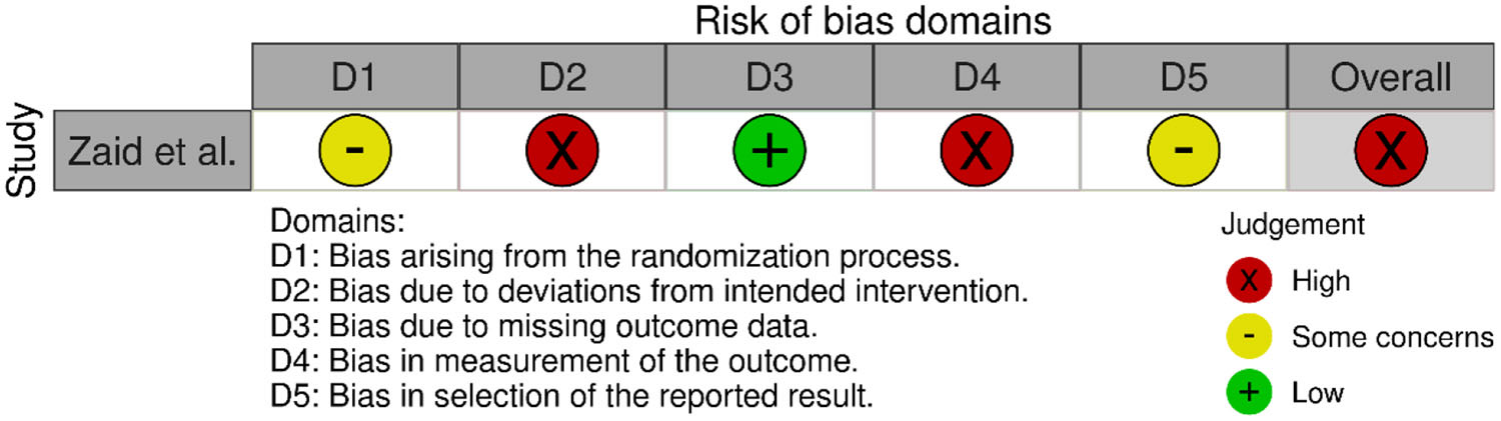
Quality assessment according to RoB2. RoB, risk‐of‐bias.

Quality assessment for the five studies was conducted using the Newcastle–Ottawa Scale (Table 2). One randomized controlled study was assessed using the RoB2 tool (Figure 2). All cohort studies included were of high quality (Newcastle–Ottawa Scale 8–9). The RCT was assessed as high risk according to the RoB2 tool.

**Table 2:** Quality assessment according to the Newcastle–Ottawa Scale.

Study	Selection	Comparability	Outcome	Total score
Zaid et al. [38]	4	2	3	9
Ebert et al. [9]	4	2	3	9
Aujla et al. [4]	4	2	2	8
Hamido et al. [13]	3	2	3	8
Zhang et al. [39]	4	2	3	9

### Αnalysis

**Figure 3:**
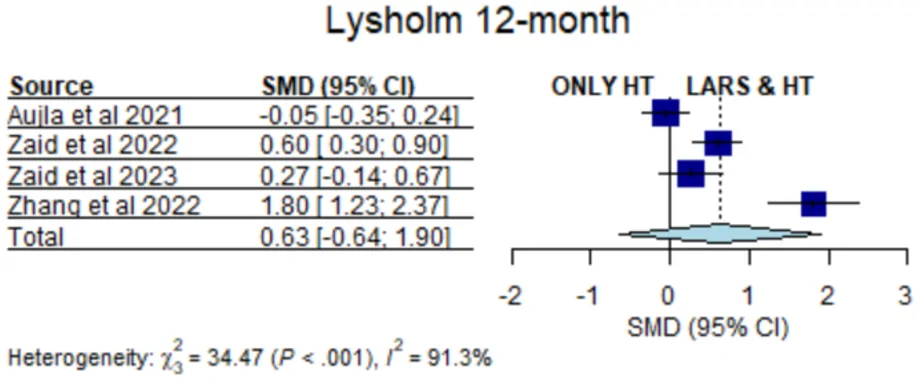
Twelve‐month Lysholm scores between LARS‐augmented and standard hamstring ACLR (pooled SMD = 0.83; 95% CI: 0.64–1.01). ACLR, anterior cruciate ligament reconstruction; CI, confidence interval; HT, hamstring tendon; LARS, Ligament Augmentation and Reconstruction System; SMD, standardized mean difference.

**Figure 4:**
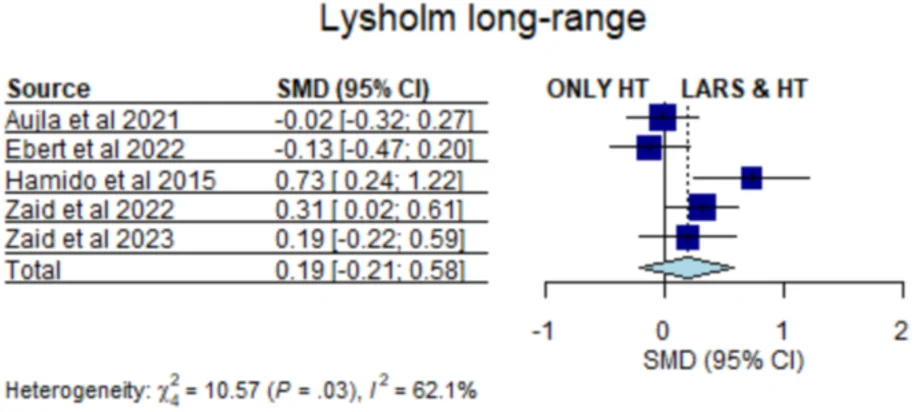
Long‐term Lysholm scores between LARS‐augmented and standard hamstring ACLR (pooled SMD = 0.19; 95% CI: 0.22–0.58). ACLR, anterior cruciate ligament reconstruction; CI, confidence interval; HT, hamstring tendon; LARS, Ligament Augmentation and Reconstruction System; SMD, standardized mean difference.

**Figure 5:**
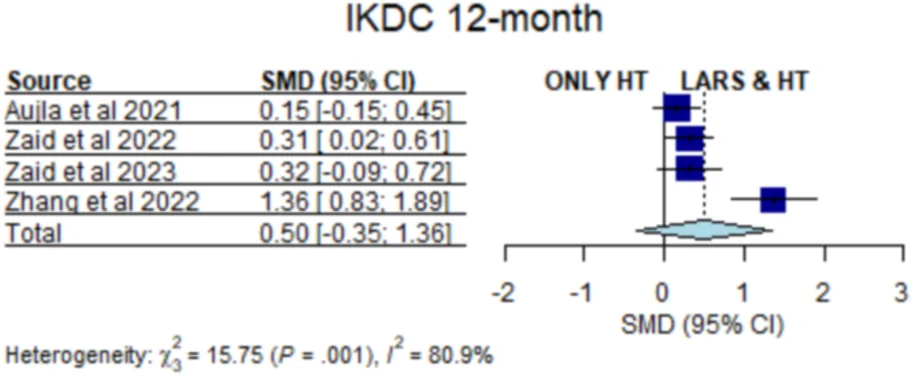
Twelve‐month IKDC scores between LARS‐augmented and standard hamstring ACLR (pooled SMD = 0.50; 95% CI: 0.35–1.36). ACLR, anterior cruciate ligament reconstruction; CI, confidence interval; HT, hamstring tendon; IKDC, International Knee Documentation Committee; LARS, Ligament Augmentation and Reconstruction System; SMD, standardized mean difference.

**Figure 6:**
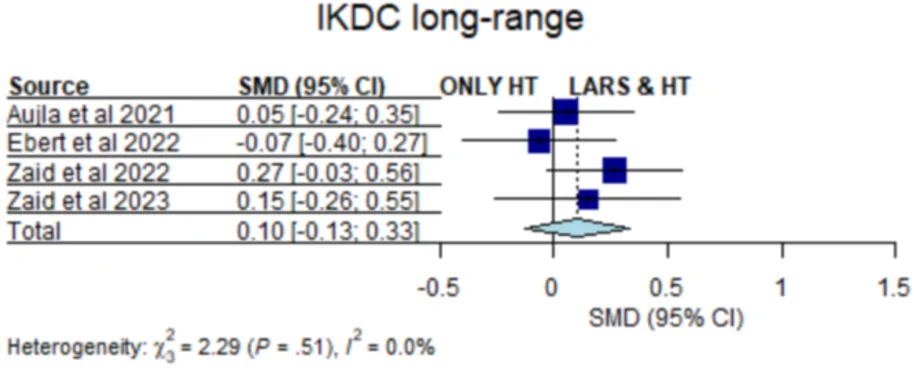
Long‐term IKDC scores between LARS‐augmented and standard hamstring ACLR (pooled SMD = 0.10; 95% CI: –0.13 to 0.33). ACLR, anterior cruciate ligament reconstruction; CI, confidence interval; HT, hamstring tendon; IKDC, International Knee Documentation Committee; LARS, Ligament Augmentation and Reconstruction System; SMD, standardized mean difference.

The pooled analysis of 12‐month Lysholm scores showed an early functional advantage for the LARS augmentation reconstructions group (SMD = 0.83, 95% confidence interval [CI]: 0.64–1.01, *p* < 0.001; *I* ^2^ = 0%; Figure 3). At ≥24 months, the difference decreased (SMD = 0.19, 95% CI: –0.21 to 0.58, *p* = 0.29; Figure 4), which suggests that the long‐term outcomes between the two groups tended to converge. Also, the pooled analysis of 12‐month IKDC scores also favoured HT augmented with LARS reconstructions (SMD = 0.50, 95% CI: 0.35–1.36, *p* < 0.001; *I* ^2^ = 42%; Figure 5). Beyond 24 months, there were no significant differences between the groups (SMD = 0.10, 95% CI: –0.13 to 0.33, *p* = 0.41; Figure 6).

**Figure 7:**
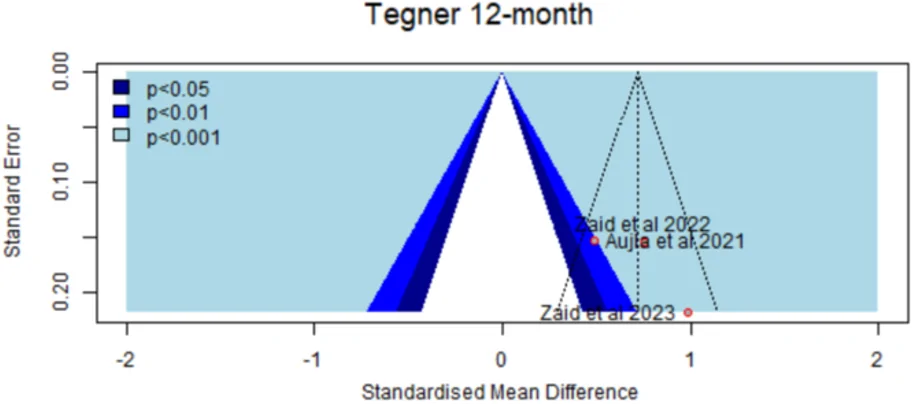
Twelve‐month Tegner scores show symmetrical distribution, suggesting insignificant risk of publication bias among the included studies.

**Figure 8:**
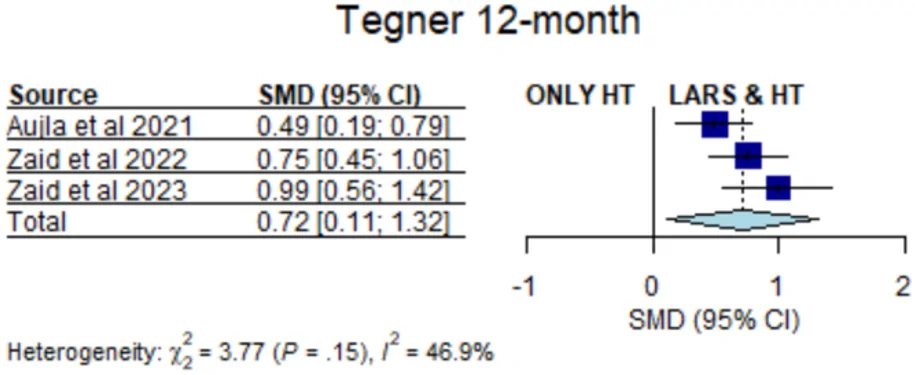
Twelve‐month Tegner scores between LARS‐augmented and standard hamstring ACLR (pooled SMD = 0.72; 95% CI: 0.11–1.32). ACLR, anterior cruciate ligament reconstruction; CI, confidence interval; HT, hamstring tendon; LARS, Ligament Augmentation and Reconstruction System; SMD, standardized mean difference.

**Figure 9:**
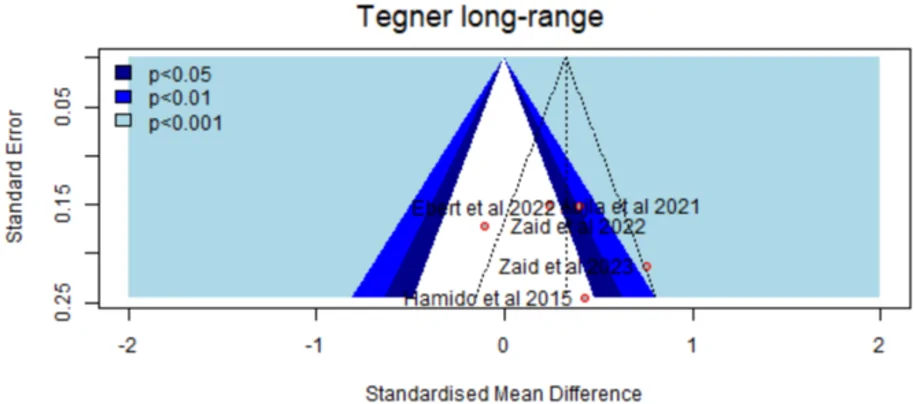
Long‐term Tegner scores show a relatively symmetrical distribution of studies, indicating a low likelihood of publication bias in the analysis.

**Figure 10:**
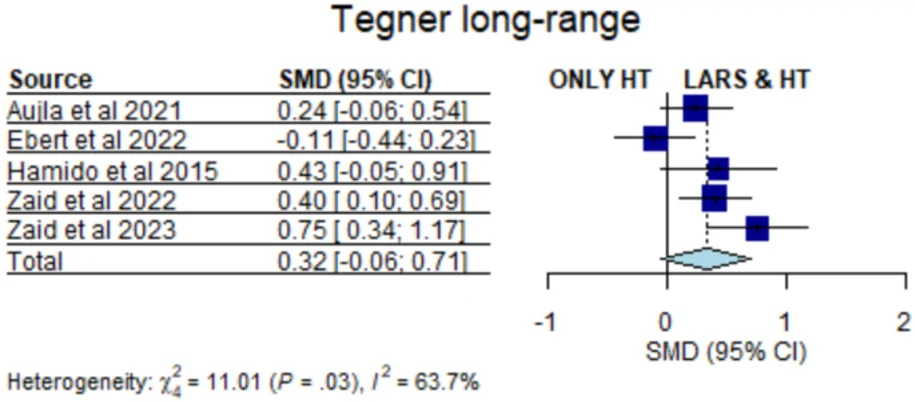
Long‐term Tegner scores between LARS‐augmented and standard hamstring ACLR (pooled SMD = 0.32; 95% CI: 0.06–0.71). ACLR, anterior cruciate ligament reconstruction; CI, confidence interval; HT, hamstring tendon; LARS, Ligament Augmentation and Reconstruction System; SMD, standardized mean difference.

Short‐term postoperative Tegner scores (12 months) were higher in the LARS augmentation reconstructions group (SMD = 0.72, 95% CI: 0.11–1.32, *p* = 0.02; *I* ^2^ = 33%; Figures 7 and 8). At long‐term follow‐up of 24 months or more, a modest but statistically significant advantage persisted for the augmented group (SMD = 0.32, 95% CI: 0.06–0.71, *p* = 0.04; Figures 9 and 10). Importantly, no study reported inferior activity levels in patients who underwent HT with LARS reconstruction. All studies reported comparable or superior patient satisfaction in the augmented group.

At 12 months, LARS augmentation showed superior outcomes compared to HT‐only grafts across several measures: greater pain relief (SMD = 0.33, 95% CI: 0.24–0.37, *I* ^2^ = 0%), higher KOOS–Symptoms scores (SMD = 0.51, 95% CI: 0.14–0.87, *I* ^2^ = 55.9%), better KOOS–ADL (activities of daily living) scores (SMD = 0.61, 95% CI: 0.28–0.95, *I* ^2^ = 56.2%), improved KOOS–Sport/Recreation scores (SMD = 0.74, 95% CI: 0.33–1.15, *I* ^2^ = 0%), and enhanced KOOS–QOL (quality of life) scores (SMD = 0.65, 95% CI: 0.22–1.07, *I* ^2^ = 25.9%) (Supporting Information S1: Appendix S2).

However, at long‐term follow‐up, none of these differences remained statistically significant—pain (SMD = 0.13, 95% CI: –0.24 to 0.37, *I* ^2^ = 0%), KOOS–Symptoms (SMD = 0.13, 95% CI: –0.17 to 0.42, *I* ^2^ = 70.5%), KOOS–ADL (SMD = 0.12, 95% CI: –0.21 to 0.45, *I* ^2^ = 74.1%), KOOS–Sport/Recreation (SMD = 0.23, 95% CI: –0.22 to 0.68, *I* ^2 ^= 23.7%), and KOOS–QOL (SMD = 0.13, 95% CI: –0.17 to 0.42, *I* ^2^ = 15.9%)—indicating convergence of functional and quality‐of‐life outcomes between the two groups over time (Supporting Information S1: Appendix S2).

Across the studies, objective anterior knee stability measured by KT‐1000 arthrometry showed no significant differences between the LARS augmented group and the HT group, confirming comparable integrity. The studies did not use the same measurement, making it difficult to categorize the results. The Hamido et al. study was the only study showing a measurable difference [13]. This study reported that the mean side‐to‐side difference was 1.1 ± 0.3 mm in the LARS group and 2.5 ± 0.5 mm in the HT group (*p* = 0.013) [13]. A side‐to‐side difference of less than 3 mm was observed in 24 patients (92%) in the LARS group and 32 patients (71%) in the HT group [33]. These findings indicate that the LARS group demonstrated significantly reduced anterior displacement compared to the HT group. Due to heterogeneous reporting of KT‐1000 values, we did not perform a meta‐analysis for this outcome.

The LARS augmentation group did not increase the overall complication rate compared with the HT group (Table 3). Total complication rates ranged from 0% to 35.8% in the augmented group and 4.4% to 27.5% in the HT group, with no statistically significant differences reported. Re‐ruptures and graft failures were uncommon, most often related to technical issues, such as femoral tunnel malposition, rather than to the synthetic material itself. Magnetic resonance imaging (MRI) and arthroscopic evaluations showed no synovitis, infection or graft‐related degeneration, and even observed fewer postoperative stiffness events and faster recovery in the augmented group.

**Table 3:** Complication between LARS groups and Hamstring graft groups.

	Total complications (LARS group)	Percentage (LARS group)	Total complication (HT group)	Percentage (HT group)
Zaid et al. [37]	6	6.7%	10	11.2%
Ebert et al. [9]	24	35.8%	19	27.5%
Zaid et al. [38]	9	19.1%	12	25.5%
Aujla et al. [4]	5	7.6%	10	7.7%
Zhang et al. [39]	5	13.8%	8	25%
Hamido et al. [13]	0	0%	2	4.4%

Abbreviations: HT, hamstring tendon; LARS, Ligament Augmentation and Reconstruction System.

HT with LARS demonstrated superior psychological readiness to resume sport during the early postoperative period. Pooled analysis showed significantly higher ACL‐RSI scores in the augmented group at 6 months (SMD = 0.45, 95% CI: 0.12–0.79, *p* = 0.007) and 12 months (SMD = 0.80, 95% CI: 0.33–1.27, *p* < 0.001) compared with the HT group (Supporting Information S1: Appendix S2). These findings demonstrate the higher early return‐to‐sport rates achieved in the hybrid group, while at longer follow‐up, psychological readiness levels between groups equalized, suggesting that LARS augmentation mainly accelerates early confidence and motivation without affecting long‐term outcomes. A summary of patient demographics, PROMs and RTS rates is presented for the autograft groups and hybrid LARS groups in Tables 4 and 5 accordingly.

**Table 4:** Summary of patient demographics, follow‐up durations, clinical scores (IKDC, KOOS, Lysholm, Tegner) and return to sport rates across studies with standard hamstring ACL reconstructions.

Study	Patients	Age (years)	Gender (M/F)	Follow‐up	IKDC (0–100)	KOOS (0–100)	Lysholm	Tegner	RTS
Zaid et al. [38]	89	27.5 ± 5.8	60/29	2 years	93.1 ± 3.0	Pain: 95.4 ± 6.0	94.1 ± 2.8	7.0 ± 1.2	86.3% (69 of 80 patients)
						Symptoms: 93.3 ± 6.8			
						ADL: 95.2 ± 7.4			
Ebert et al. [9]	69	30.8 (16–49)	44/25	7.7 years (7–9.5)	85.5 (40.2–100.0)	Not specifically mentioned	89.5 (10.9, 53–100)	6.0 (3–10)	53 patients (76.8%)
Zaid et al. [37]	47	26.1 ± 4.0	35/12	3–8 years	91.0 ± 3.8	90.0 ± 4.3	92.4 ± 4.1	6.0 ± 1.1	75.6% (31/41 pt)
Aujla et al. [4]	130	27.5 ± 8.6	79/51	2 years	91.2 ± 9.4	Pain: 95.9 ± 6.0	94.0 ± 8.5	7.0 ± 1.7	113 (86.9%)
						Symptoms: 93.9 ± 7.2			
						ADL: 99.1 ± 2.3			
						Sport/Rec: 91.3 ± 8.2			
						QoL: 78.9 ± 18.8			
Zhang et al.[39]	32	33 (21–42)	24/8	12 months	93.7 ± 2.1	Not specifically mentioned	94.6 ± 1.7	6.9 (value unclear)	Not explicitly mentioned
Hamido et al. [13]	45	20 (18–31)	44/1	≥5 years (58–62 mo)	Pre‐op: Severely abnormal in 28	KOOS–Pain: 81.2	Pre: 42.3 ± 5.2	Pre: 3.3 ± 0.3	Not explicitly mentioned
					2 years: Normal in 32, nearly normal in 7	KOOS–Symptoms: 78.2	Post: 90.1 ± 6.9	Post: 6.7 ± 1.5	
					5 years: Normal in 26, nearly normal in 6	KOOS–ADL: 92.0			

Abbreviations: ACL, anterior cruciate ligament; ADL, activities of daily living; HT, hamstring tendon; IKDC, International Knee Documentation Committee; KOOS, Knee injury and Osteoarthritis Outcome Score; LARS, Ligament Augmentation and Reconstruction System; RTS, return to sport.

**Table 5:** Summary of patient demographics, follow‐up durations, clinical scores (IKDC, KOOS, Lysholm, Tegner), return to sport rates and complications across studies with LARS augmented ACL reconstructions.

Study	Patients	Age (years)	Gender (M/F)	Follow‐up	IKDC (0–100)	KOOS (0–100)	Lysholm	Tegner	RTS
Zaid et al. [38]	89	26.2 ± 4.7	65/24	2 years	94.0 ± 3.7	Pain: 96.1 ± 6.2	95.1 ± 3.5	7.5 ± 1.3	97.4%
						Symptoms: 94.0 ± 7.2			
						ADL: 96.7 ± 8.3			
Ebert et al. [9]	67	31.1 (16–49)	45/22	7.9 years (7–10)	84.6 (SD 13.2, 51.7–100.0)	Not specifically mentioned	87.9 (range 44–100)	5.8 (3–10)	77.6%
Zaid et al. [37]	47	26.4 ± 3.5	33/14	3–8 years	91.6 ± 4.2	89.3 ± 5.4	93.2 ± 4.3	6.8 ± 1.0	85.4% in 24 months
Aujla et al. [4]	66	26.8 ± 9.5	44/22	2 years	91.7 ± 8.5	Pain: 95.5 ± 7.3	93.8 ± 7.8	7.4 ± 1.6	87.9%
						Symptoms: 92.8 ± 8.7			
						ADL: 97.1 ± 7.8			
						Sport: 89.4 ± 14.0			
						QoL: 82.6 ± 16.6			
Zhang et al. [39]	36	27 (17–37)	30/5	12 months	96.9 ± 2.5	Not specifically mentioned	97.9 ± 1.9	7.1 (not exact)	Not explicitly mentioned
Hamido et al. [13]	27	24 (21–35)	27/0	≥5 years (58–62 mo)	Pre‐op: 17 severely abnormal	Pain: 84.6	Pre: 43.6 ± 3.6	Pre: 3.6 ± 0.7	Not explicitly mentioned
					2 years: 21 normal, 5 nearly normal	Symptoms: 86.3	5 years: 95.3 ± 7.3	5 years: 7.4 ± 1.8	
					5 years: 20 normal, 6 nearly normal	ADL: 94.2			

Abbreviations: ACL, anterior cruciate ligament; ADL, activities of daily living; HT, hamstring tendon; IKDC, International Knee Documentation Committee; KOOS, Knee injury and Osteoarthritis Outcome Score; LARS, Ligament Augmentation and Reconstruction System; RTS, return to sport.

## DISCUSSION

This systematic review and meta‐analysis compared outcomes of ACL reconstruction using hamstring autografts (HT) versus hamstrings augmented with LARS. Across all studies, LARS augmentation consistently improved early postoperative recovery—particularly subjective function, activity levels, psychological readiness and return‐to‐sport (RTS)—without increasing complications or compromising long‐term graft integrity.

### Patient‐reported outcome measures

LARS provided clear short‐term advantages in Lysholm, IKDC, Tegner, KOOS, and ACL‐RSI scores during the first 6–12 months [4, 9, 13, 37, 38]. These improvements reflect faster symptom relief, earlier functional gains and better confidence during the demanding early rehabilitation phase. This benefit is biologically plausible: the graft undergoes a vulnerable remodelling period between 5–9 weeks, characterized by inflammation and reduced mechanical strength [4, 38]. LARS augmentation likely reduces stress on the healing graft, helping protect early stability and supporting accelerated rehabilitation [9]. Despite meaningful early differences, functional outcomes converged after 18–24 months. Long‐term Lysholm, IKDC, Tegner, KOOS and ACL‐RSI scores were similar between the two groups, indicating that LARS accelerates early recovery but does not change ultimate outcome trajectories [4, 9, 13, 37, 38]. No study reported inferior long‐term results with LARS.

Objective knee stability was measured by KT‐1000 arthrometry or clinical examination to measure anterior tibial translation following ACL reconstruction. In studies that used KT‐1000, the LARS group showed the same or better stability compared to hamstring grafts. No significant differences in KT‐1000 side‐to‐side measurements were observed, indicating mechanical equivalence [9, 13, 37, 38]. In studies that used clinical tests like Lachman and Pivot Shift, the LARS group showed better results early after surgery [4, 39]. But these differences became smaller and usually disappeared by 12–18 months. Very important, no study showed worse anterior knee stability in the LARS group. This supports that LARS is a safe and reliable technique, both in short‐term and long‐term follow‐up.

Radiographic and MRI‐based follow‐up found no statistically important differences between the LARS‐augmented and hamstring groups in graft signal intensity, tunnel widening, synovitis or osteoarthritic progression [38, 39]. Long‐term, including WORMS scoring at 7 years, report absence of increased degenerative changes or synthetic graft‐related complications [38]. Clinical outcomes showed satisfactory joint function, with reduced stiffness and crepitus in LARS groups during early recovery [9]. No study found any radiological complications caused by the LARS graft. These results show that LARS is safe for the joint, and the surgical technique (tunnel placement) is more important for long‐term success than the graft type alone.

The second‐look arthroscopy was used to directly check the graft healing (morphology), tension, synovial coverage, and how well it was accepted by the body after ACL reconstruction. Only Zaid et al. did structured comparisons using this method [37]. Their results showed that LARS‐augmented grafts had similar tension, position, and synovial coverage as the standard hamstring grafts [37]. There were no full graft failures or serious inflammation, and partial tears were very few [39]. Also, no signs of foreign body reaction, fibrosis, or poor healing were seen in the LARS group [37]. This means the synthetic part did not affect the biological healing process and the claim that synthetic LARS augmentation might cause inflammation does not hold any value, at least in the context of ACLR.

In all studies, the LARS group was related to faster functional recovery and higher RTS rates in the early postoperative period, mainly within the first 6–12 months (Tables 4 and 5). The LARS group reported better short‐term functional outcomes compared to those with standard hamstring autografts. These early benefits were clearer in high‐demand populations, like athletes [4, 9, 37, 38]. More LARS patients are returning to Level 1 activities and preinjury sport levels within 1 year. Conversely, by 18–24 months postoperatively, functional outcomes and RTS rates were similar in both groups, with no significant long‐term differences observed [4, 9, 37, 38]. No study showed inferior functional or RTS results with LARS. These results support that LARS augmentation is a choice for faster rehabilitation and early RTS, particularly in active individuals, while still giving the same long‐term results as normal graft techniques [4, 9, 37, 38].

In all the studies, LARS augmentation did not show more complications, which means it is a safe method for ACL reconstruction (Table 2). The synthetic ligaments had low rates of synovitis, infection, tunnel widening, and graft failure, with complications like those of standard hamstring autografts [4, 9, 13, 37, 38]. The ACL retear rates were between 1.3% and 11.3% across studies, with no statistically significant differences between LARS and non‐LARS groups. Failure events were most often because of technical errors, such as femoral tunnel malposition, rather than the use of synthetic augmentation itself. MRI and arthroscopic evaluations showed no evidence of inflammatory reactions or degenerative joint changes connected to LARS. Some studies noted fewer postoperative stiffness and earlier recovery in the LARS group, without increased adverse events. The limited current evidence suggests that LARS augmentation provides a mechanically and biologically safe option, optimizing early postoperative outcomes without endangering long‐term stability or complications, although further high‐quality studies are needed to fully understand its role in ligament reconstruction.

Our analysis yields similar results with prior studies reporting quicker improvements in clinical scores and functional capacity in the early rehabilitation phase for LARS‐augmented grafts. Zaid et al. established significantly better Lysholm, IKDC and ACL‐RSI scores at early follow‐up in the LARS group with faster return to pivoting sports [37]. Similarly, Aujla et al. and Zhang et al. found higher Tegner and IKDC scores in the LARS‐augmented group at 1 year, suggesting a potential role for LARS augmentation during the demanding early healing stage of the graft [4, 39]. Ebert et al. reported similar outcomes, including KT‐1000 laxity, WORMS scores and complication rates, between double‐bundle hamstring reconstructions with or without LARS augmentation at 7‐year follow‐up [9]. Furthermore, in our review, we also did not find any increase in failure or re‐tear rates. Hamido and Zaid also showed that LARS helped patients with small grafts or very active lifestyle [13, 38]. Even though the early outcomes are better with LARS, long‐term results like KT‐1000 and second‐look arthroscopy showed no big differences. This means LARS helps early rehabilitation but does not negatively affect healing or the graft's strength over time. Zaid et al. also confirmed this in their second‐look findings [37, 38].

The LARS augmented grafts showed earlier improvement, but the clinical advantages seemed to be met with traditional hamstring grafts beyond 18–24 months postoperatively. This means that LARS mainly helps in the early healing period, but it does not really change the final long‐term results. There are also some limits in this review. Even if most studies were prospective and comparative, there were differences in surgical technique, graft fixation methods and rehabilitation protocols leading to heterogeneity. Additionally, in many cases, the choice to use LARS was not random, which may introduce selection bias. The number of studies included, despite high quality, was small, leading to underpowered results, especially in funnel plots assessing publication bias. Our choice of PubMed and Scopus as our databases also suggests a suboptimal condition and limitation according to the Cochrane methodology. We acknowledge that conducting multiple comparisons without statistical adjustment theoretically increases the risk of Type I error (false positives) [30]. However, we observed statistically significant differences favouring the LARS group across all distinct patient‐reported outcome measures (IKDC, KOOS, Lysholm, Tegner) consistently at the 6‐ and 12‐month follow‐ups. Since these instruments measure related constructs yet are mathematically distinct, the probability of obtaining uniform false‐positive results across every scale simultaneously is low. This consistent pattern suggests a robust clinical signal rather than statistical noise [6], reinforcing the validity of the early postoperative benefits observed. Finally, additional randomized studies with similar rehabilitation protocols, along with subgroup analysis (poor graft diameter, high body mass index [BMI] patients), are needed to definitively evaluate the benefits of LARS augmentation in various patient populations.

## CONCLUSIONS

Hamstrings autograft augmented with LARS seems to provide superior early PROMs and psychological readiness to RTS, in comparison to traditional standalone hamstring autograft. The stiff nature of the synthetic graft might be implicated in this, providing a safer and more stable feel for the patient in the early postoperative period. Long‐term outcomes and complication rates were found to be similar to the hamstrings group, in contrast to older studies implicating synthetic grafts with rupture rates and synovitis. Newer, biocompatible generations of artificial ligaments and the addition of hamstring autograft might be responsible for this. More and higher‐quality prospective studies are needed to fully understand the implications of hybrid LARS augmented grafts in ligament reconstruction. Future questions include the use of hybrid grafts in suboptimal ligament quality or subgroups, including older or higher BMI patients and athletes requiring early RTS.

## AUTHOR CONTRIBUTIONS

**Panagiotis Antzoulas**: Writing original draft; investigation; data curation; methodology. **Vasileios Giannatos**: Writing—review and editing; investigation; methodology. **Andreas Panagopoulos**: Project administration; supervision. **John Lakoumentas**: Visualization; formal analysis; data curation. **Vasileios Athanasiou**: Investigation. **Antonios Kouzelis**: Verification. **Zinon Kokkalis**: Supervision. **Irini Tatani**: Resources; investigation. **Spyridon Plesas:** Supervision. **John Gliatis**: Conceptualization; supervision; project administration.

## CONFLICT OF INTEREST STATEMENT

The authors declare no conflicts of interest.

## ETHICS STATEMENT

The authors have nothing to report.

## Supporting information

Appendix 1 – Search Strategy. Appendix 2. Figure 1: Forest plots comparing KOOS–Pain scores at 12‐month and long‐term follow‐ups. Figure 2: Analysis of KOOS–Symptoms subscale outcomes at 12 months and long‐term follow‐up. Figure 3: KOOS–ADL Functional Performance at 12 Months and Long‐Term, assessment of Activities of Daily Living (KOOS–ADL. Figure 4: Forest plots showing KOOS‐Sport/Recreation scores at 12 months and long‐range. Figure 5: Analysis of KOOS–QOL scores (12‐Month and Long‐Term Outcomes). Figure 6: Forest plot comparing 6‐month ACL‐RSI scores for LARS‐augmented vs hamstring‐only ACL reconstruction. Figure 7: Funnel plot assessing publication bias in studies evaluating 6‐month ACL‐RSI scores. Figure 8: Forest plot comparing 12‐month ACL‐RSI scores for LARS‐augmented vs hamstring‐only ACL reconstruction.

## Data Availability

The data that support the findings of this study are available from the corresponding author upon reasonable request.
