# Supplementary material for: LARS‐augmented hamstring ACL reconstruction shows better early but similar long‐term outcomes compared with hamstring autograft alone: A systematic review and meta‐analysis
Source: J Exp Orthop. 2026 Feb 24;13(1):e70654. doi: 10.1002/jeo2.70654 (PMC12930285; doi:10.1002/jeo2.70654)
Supplement: Supplementary file 1 — Appendix 1 – Search Strategy. Appendix 2. Figure 1: Forest plots comparing KOOS–Pain scores at 12‐month and long‐term follow‐ups. Figure 2: Analysis of KOOS–Symptoms subscale outcomes at 12 months and long‐term follow‐up. Figure 3: KOOS–ADL Functional Performance at 12 Months and Long‐Term, assessment of Activities of Daily Living (KOOS–ADL. Figure 4: Forest plots showing KOOS‐Sport/Recreation scores at 12 months and long‐range. Figure 5: Analysis of KOOS–QOL scores (12‐Month and Long‐Term Outcomes). Figure 6: Forest plot comparing 6‐month ACL‐RSI scores for LARS‐augmented vs hamstring‐only ACL reconstruction. Figure 7: Funnel plot assessing publication bias in studies evaluating 6‐month ACL‐RSI scores. Figure 8: Forest plot comparing 12‐month ACL‐RSI scores for LARS‐augmented vs hamstring‐only ACL reconstruction. [file JEO2-13-e70654-s001.docx]

**Supplementary material**

**Appendix 1 – Search Strategy**

PubMed Search Strategy:
(((ACL) OR (ANTERIOR CRUCIATE LIGAMENT)) AND (RECONSTRUCTION)) AND (LARS)

Scopus Search Strategy:
('acl' OR 'anterior cruciate ligament') AND 'reconstruction' AND 'LARS'

No publication year restriction was applied. English studies were included only.

**Appendix 2**


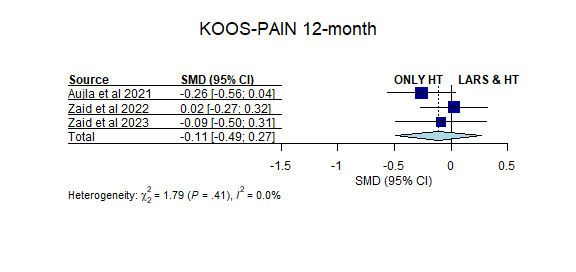

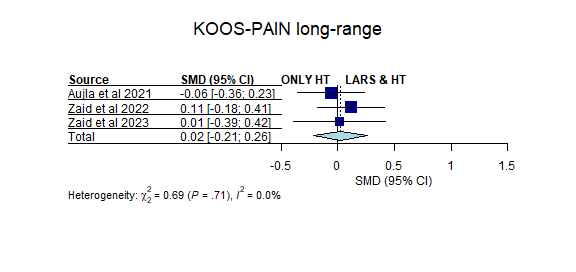


**Figure 1:** Forest plots comparing KOOS–Pain scores at 12-month and long-term follow-ups.


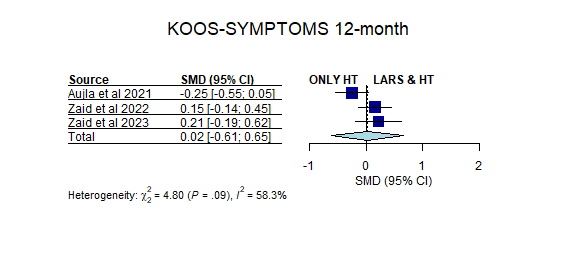

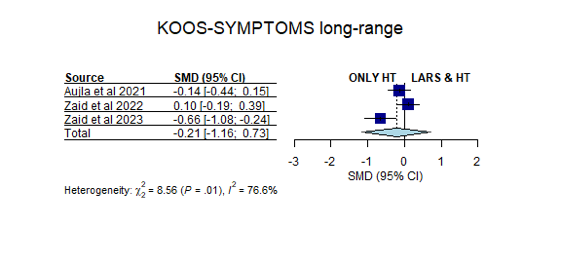


**Figure 2:** Analysis of KOOS–Symptoms subscale outcomes at 12 months and long-term follow-up.


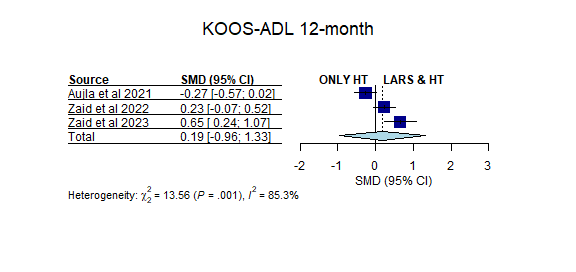

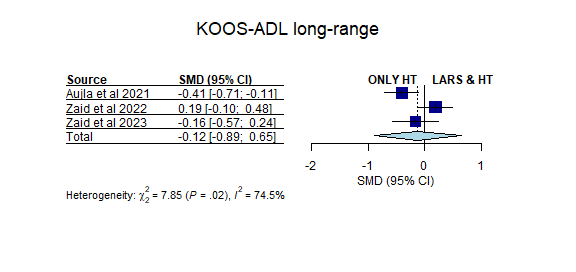


**Figure 3:** **KOOS–ADL Functional Performance at 12 Months and Long-Term**, assessment of Activities of Daily Living (KOOS–ADL).


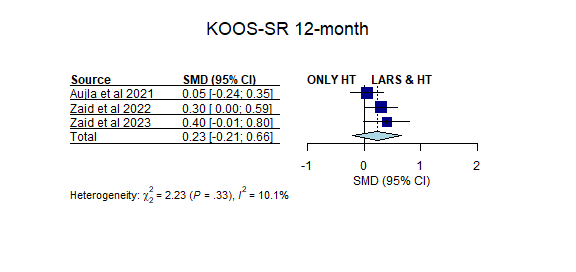

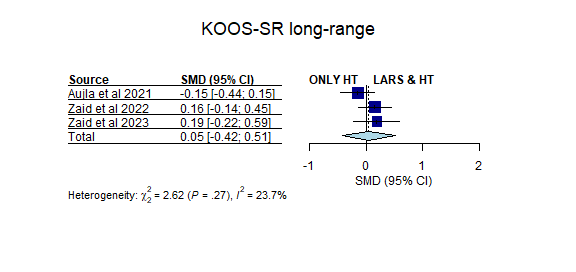


**Figure 4:** Forest plots showing KOOS-Sport/Recreation scores at 12 months and long-range.


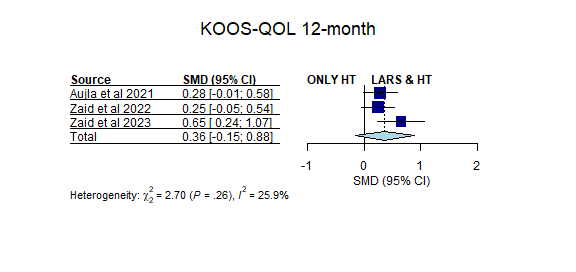

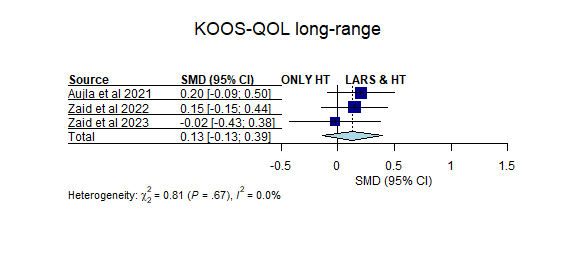


**Figure 5:** Analysis of KOOS–QOL scores (**12-Month and Long-Term Outcomes)**.


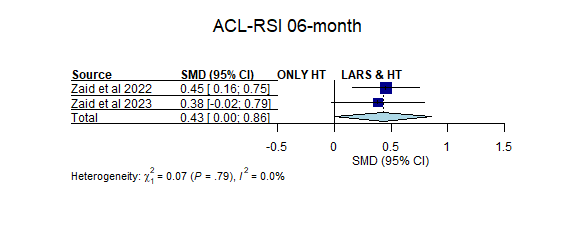


**Figure 6:** Forest plot comparing 6-month ACL-RSI scores for LARS-augmented vs hamstring-only ACL reconstruction.


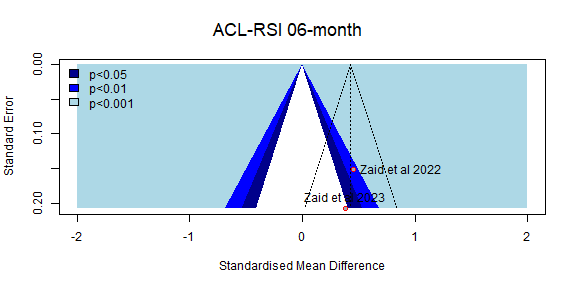


**Figure 7:** Funnel plot assessing publication bias in studies evaluating 6-month ACL-RSI scores.


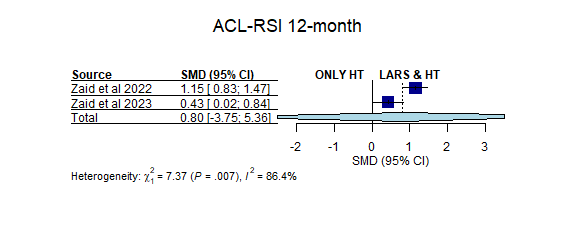


**Figure 8:** Forest plot comparing 12-month ACL-RSI scores for LARS-augmented vs hamstring-only ACL reconstruction.
